# Spinterface Effects in Hybrid La_0.7_Sr_0.3_MnO_3_/SrTiO_3_/C_60_/Co Magnetic Tunnel Junctions

**DOI:** 10.1021/acsaelm.2c00300

**Published:** 2022-08-24

**Authors:** Ilaria Bergenti, Takeshi Kamiya, Dongzhe Li, Alberto Riminucci, Patrizio Graziosi, Donald A. MacLaren, Rajib K. Rakshit, Manju Singh, Mattia Benini, Hirokazu Tada, Alexander Smogunov, Valentin A. Dediu

**Affiliations:** † Institute of Nanostructured Materials ISMN-CNR, Via Gobetti 101, Bologna 40129, Italy; ‡ Department of Materials Engineering Science, Osaka University, 1-3, Machikaneyama, Toyonaka, Osaka, Japan 560-8531; § CEMES, Université de Toulouse, CNRS, 29 rue Jeanne Marvig, F-31055 Toulouse, France; ∥ SUPA, School of Physics and Astronomy, University of Glasgow, Glasgow G12 8QQ, U.K.; ⊥ CSIR - National Physical Laboratory, Dr. K. S. Krishnan Marg, New Delhi 110012, India; # Service de Physique de l’Etat Condensé (SPEC), CEA, CNRS, Université Paris-Saclay, CEA Saclay, 91191 Gif-sur-Yvette Cedex France

**Keywords:** tunnel junction, spinterface, molecular spintronics, C_60_, hybrid interface, spin-dependent density of states

## Abstract

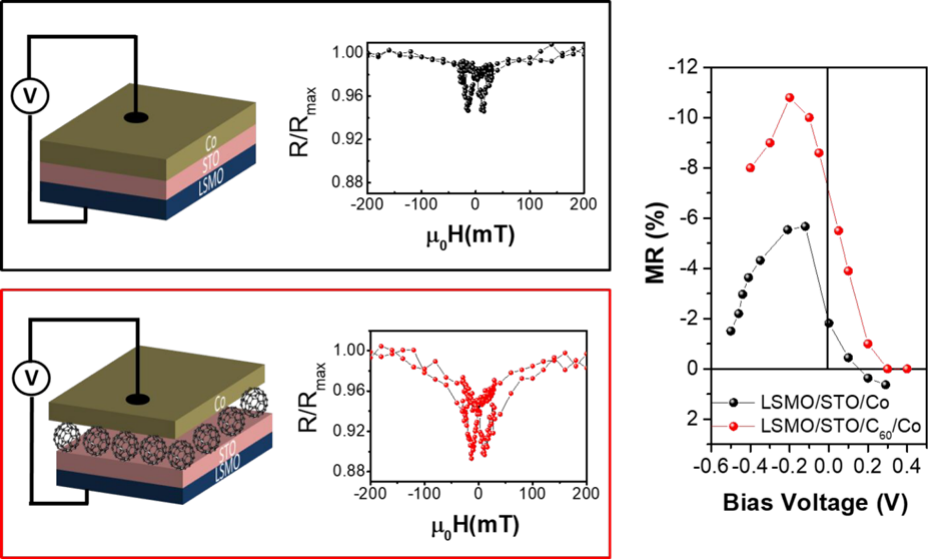

Orbital hybridization at the Co/C_60_ interface been has proved to strongly enhance the magnetic anisotropy of the cobalt layer, promoting such hybrid systems as appealing components for sensing and memory devices. Correspondingly, the same hybridization induces substantial variations in the ability of the Co/C_60_ interface to support spin-polarized currents and can bring out a spin-filtering effect. The knowledge of the effects at both sides allows for a better and more complete understanding of interfacial physics. In this paper we investigate the Co/C_60_ bilayer in the role of a spin-polarized electrode in the La_0.7_Sr_0.3_MnO_3_/SrTiO_3_/C_60_/Co configuration, thus substituting the bare Co electrode in the well-known La_0.7_Sr_0.3_MnO_3_/SrTiO_3_/Co magnetic tunnel junction. The study revealed that the spin polarization (SP) of the tunneling currents escaping from the Co/C_60_ electrode is generally negative: i.e., inverted with respect to the expected SP of the Co electrode. The observed sign of the spin polarization was confirmed via DFT calculations by considering the hybridization between cobalt and molecular orbitals.

## Introduction

The formation of a hybridized layer at the interface between ferromagnetic metals and organic semiconductors has been proven to be efficient in the modulation of the magnetic and spin properties of both components.^1,2^ An illustrative example is represented by the interface between the pure carbon allotrope buckminsterfullerene (C_60_) and ferromagnetic 3d metals,^3−5^ such as Co. Orbital hybridization at the interface between a C_60_ overlayer and an epitaxial ultrathin film of Co(0001) leads to a considerable change in the magnetic anisotropy of the Co,^6^ able to induce a magnetization reorientation transition from in-plane to out-of-plane in the ferromagnetic layer and a magnetic hardening.^7^ Correspondingly, the nonmagnetic C_60_ molecule is also modified,^8^ becoming spin active by acquiring a net magnetic moment as a consequence of coupling of the carbon atoms closest to the underlying Co.

These unusual features liven up the Co/C_60_ interface as a fascinating element for the modulation of the spin functionality in solid-state devices: the magnetic hardening of a FM layer, obtained by interfacing it with an organic molecule, is of technological interest in spintronic memories^9^ and also the induced spin selectivity at the interface is fundamental for the spin injection.^10^ In this regards, spin transport has been proved to depend on the coupling of C_60_ molecules on the magnetic surface in case of fcc-Co(111)/C_60_^11^ and Cr(001)/C_60_.^12^

Here we prove that Co/C_60_ interface can be integrated in the prototypical magnetic tunnel junction (MTJ) La_0.7_Sr_0.3_MnO_3_/SrTiO_3_/Co (LSMO/STO/Co),^13^ resulting in a change of the spin tunneling current of the device. We address this issue by inserting an ultrathin C_60_ molecular layer at the interface with the ferromagnetic Co layer acting as a spin-polarized electrode and by comparing the magnetotransport properties of LSMO/STO/C_60_/Co MTJ with those of the reference LSMO/STO/Co MTJ. Given that spin-polarized tunneling in an MTJ depends on the band structure of the insulating barrier, on the properties of ferromagnetic layers, and even more critically on those of their interfaces, an investigation of TMR values and sign together with a calculation of spin conductance in an MTJ thus provides a direct method to test the spin polarization at the C_60_/Co interface, allowing unlocking of potential applications of the Co/C_60_ interface in solid-state spintronic devices.

## Experimental Methods

### Experiments

**Figure 1:**
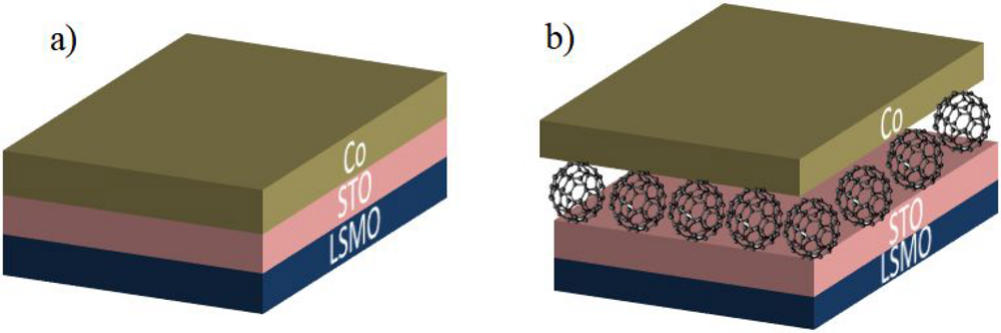
Schematic drawings of MTJ junctions: (a) reference device La_0.7_Sr_0.3_MnO_3_/SrTiO_3_/Co; (b) C_60_-seeded MTJ.

Cross-bar LSMO/STO/Co and LSMO/STO/C_60_/Co were obtained by a shadow-masking technique on single-crystal NdGaO_3_ (NGO) (110) substrates. An LSMO layer that was 15 nm thick was deposited by a channel spark ablation method following the procedure described by Graziosi et al.^14^ The STO tunneling barrier (5 nm) was grown with the same CSA technique under an O_2_ atmosphere (10^–2^ mbar), keeping the substrate at 700 °C. The Co top contact was obtained by e-gun evaporation under UHV (*P* < 10^–9^ mbar) at room temperature. The 2 nm thick C_60_ layer was deposited on STO by thermal evaporation with a ultralow flux MBKomponente cell with a growth rate of 0.15 Å/s. The Co top contact was obtained by e-gun evaporation under UHV (*P* < 10^–9^ mbar) at room temperature. C_60_ was not damaged by the fabrication of the top Co electrode.^15^ Metallic contacts were provided by gold pads evaporated on the LSMO and the Co electrodes. Junctions were 500 μm × 500 μm in size. The overall structures are depicted in Figure 1.

Transmission electron microscopy (TEM) was employed for structural characterization, using a probe-corrected JEOL ARM200cF instrument that was operated at 200 kV and was equipped with a cold field emission electron gun and a Gatan Quantum electron energy loss spectrometer. Cross-sectional samples were prepared using standard “lift-out” procedures on an FEI Nova Nanolab Focused Ion Beam instrument.

The topography of C_60_ films was investigated by using an AFM Smena microscope (NT-MDT, Moscow, Russia) in noncontact mode (NCM) under ambient conditions. Silicon cantilevers were employed.

Spin transport measurements were performed using an exchange gas cryostat, equipped with an electromagnet. A van der Pauw configuration was adopted to minimize the contributions from the electrode resistances. The TMR was measured by sweeping the magnetic field in the plane of the device, while applying various constant biases through the junction by using a Keithley 2400 SMU in the temperature range 100–300 K, with a maximum applied field of μ_0_*H* = 0.9 T. The LSMO electrode was biased, while the Co was grounded.

### Density Functional Theory Calculations

We performed spin-polarized *ab initio* calculations using the plane wave electronic structure package Quantum ESPRESSO^16^ in the framework of density functional theory (DFT). We used Perdew–Burke–Ernzerhof parametrization (PBE) for exchange-correlation functionals and the ultrasoft pseudopotential formalism. Energy cutoffs of 30 and 300 Ry were employed for the wave functions and the charge density, respectively. The C_60_/ferromagnetic interface was simulated using a seven-layer slab of hcp-Co (0001) and a 4 × 4 in-plane periodicity. The full system was first relaxed, fixing four Co bottom layers at their bulk positions and using a 2 × 2 *k*-point mesh; then the electronic properties of the relaxed structure were studied using a finer 6 × 6 mesh of *k*-points. The same computational parameters as in ref (17) were used.

## Results and Discussion

**Figure 2:**
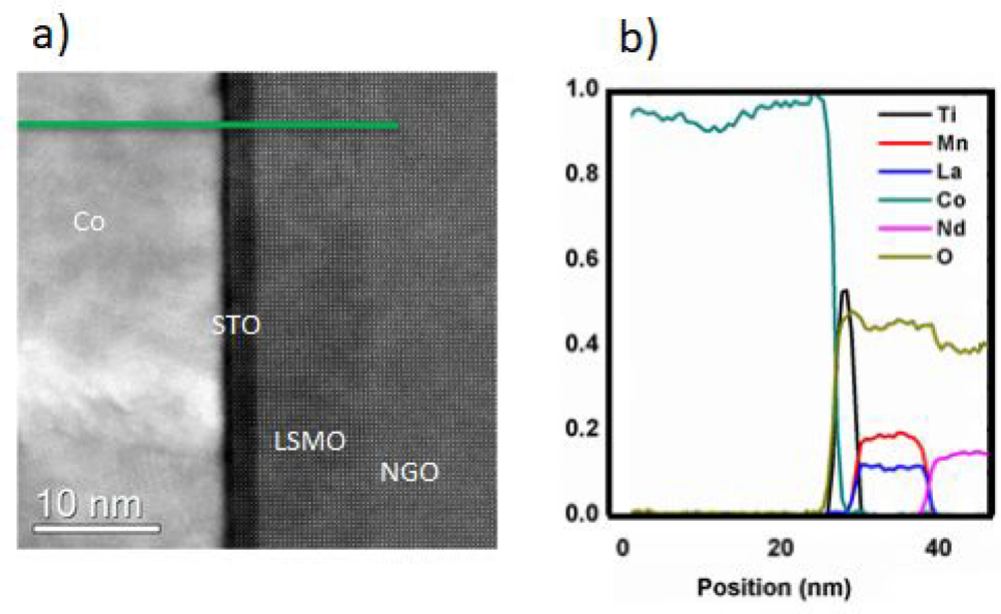
(a) TEM cross section image of the LSMO/STO/Co junction. The STO layer is completely crystalline, along with the LSMO and NGO. (b) EELS analysis of the LSMO/STO/Co junction along the green line.

A high-resolution transmission electron microscopy (HR-TEM) cross-section image of LSMO (15 nm)/STO (5 nm)/Co (50 nm) confirms the excellent morphology of the MTJ. Figure 2a clearly shows that LSMO is epitaxial with a (100) orientation over the NGO (110) substrate, as well as over the epitaxial STO layer. The Co was polycrystalline, as expected for room-temperature deposition. Images revealed an abrupt epitaxial LSMO/STO interface and a less sharp STO/Co interface, as shown by an EELS scan performed along the cross-section of the sample (Figure 2b).

**Figure 3:**
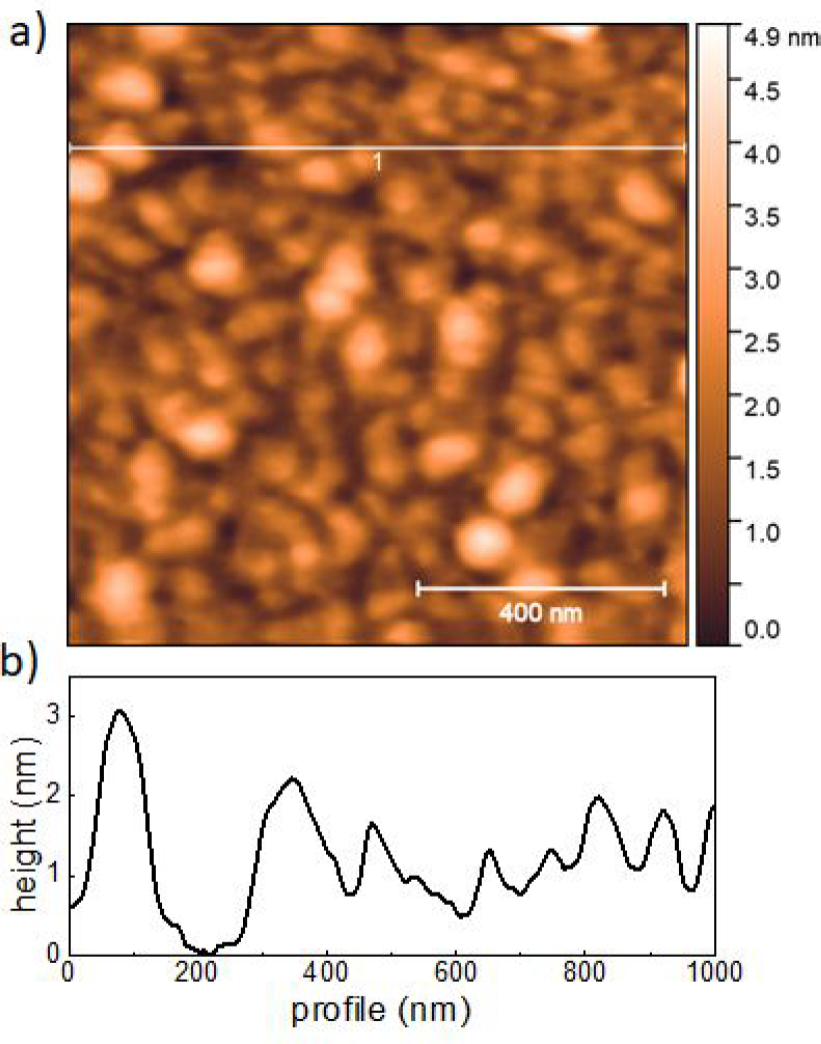
(a) Topography of a 2 nm C_60_ film grown on a STO substrate at room temperature. (b) Line profile measurement along the white line shown in (a).

The insertion of a C_60_ layer is obtained by depositing the organic layer onto the LSMO/STO. The STO tunnel barrier exhibits a smooth surface (RMS < 0.3 nm: i.e., STO lattice parameter, see section SI1 in the Supporting Information). After the growth of 2 nm C_60_, the molecules form clusters distributed on the STO surface with an overall roughness of about 1 molecular layer (RMS = 0.7 ± 0.1 nm), as shown in Figure 3. On closer inspection, AFM height profiles (see section SI1) indicate a quite uniform coverage of the surface with an estimated coverage of 98%.

Subsequently, a Co deposition was performed. The partial intermixed layer between Co and C_60_ is unavoidable, given the RMS of the C_60_ layer, but several works pointed out that the quite compact nature of the C_60_ molecule prevents the diffusion of the Co ion into the molecule.^15,18^ Moreover, during the Co growth, the C_60_ molecule maintains its integrity^19^ and molecular clusters tend to be incapsulated beneath the Co film. It is worth noting that, even in case of submonolayer deposition,^20^ a C_60_ layer has been used as buffer layer in organic light-emitting diodes to efficiently improve the qualities of the interface with metals by changing the interfacial work functions and the energy level alignment.^21^

**Figure 4:**
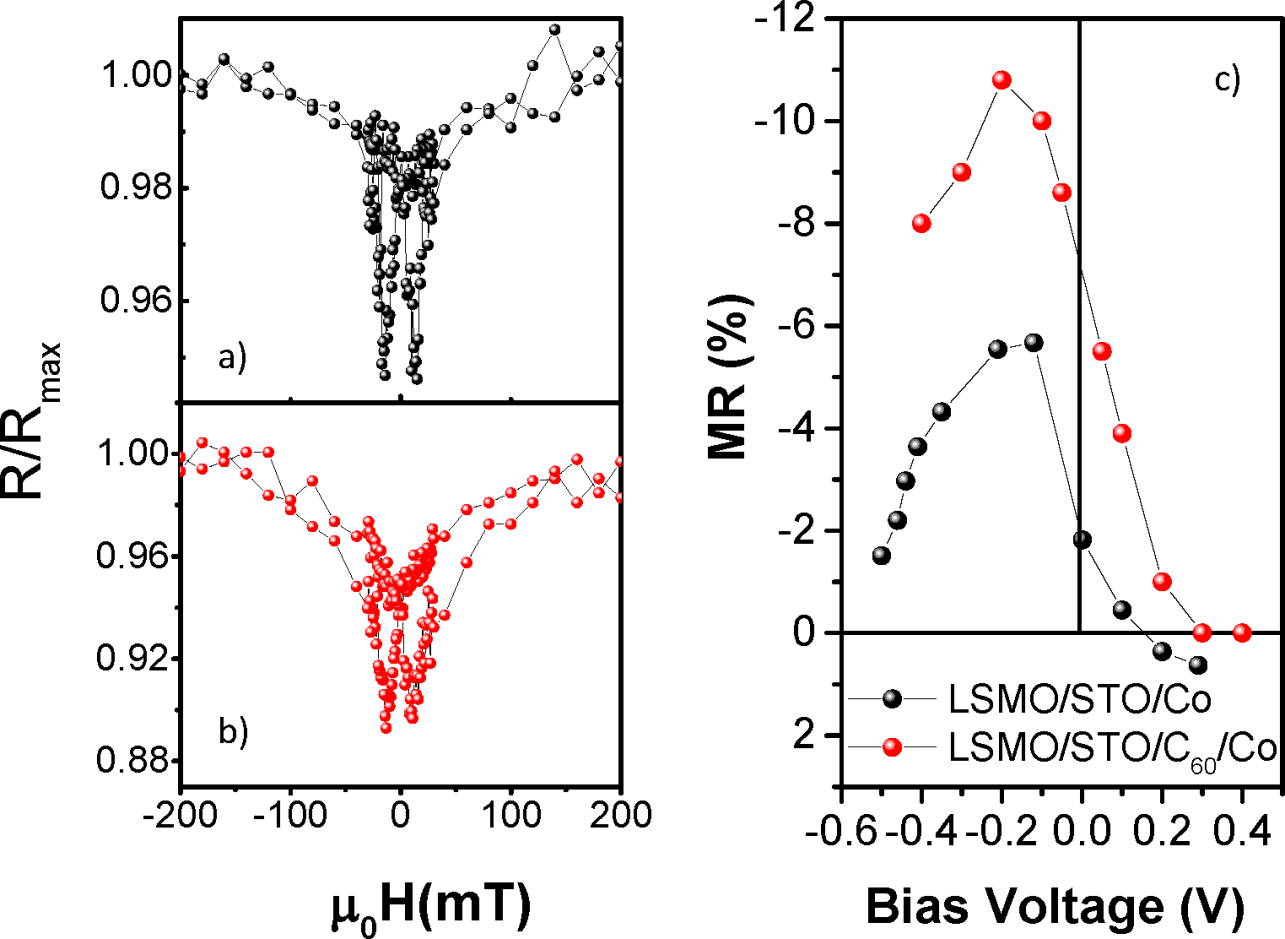
Negative tunneling magnetoresistance (TMR) as a function of applied field for the two junctions (a) LSMO/STO/Co and (b) LSMO/STO/C_60_/Co measured with a voltage bias of 100 mV. (c) TMR ratio as a function of the applied dc bias for LSMO/STO/Co junctions (black balls) and LSMO/STO/C_60_/Co (red balls). Error bars are within the symbol size.

Once the role of C_60_ as decoupling layer between Co and STO was addressed, we addressed transport data measured in the current-perpendicular-to-plane (CPP) geometry. Conduction across the LSMO/STO/Co heterostructure exhibits the typical features expected for a tunneling conduction process (see section SI2), ruling out any possible Ohmic path. The magnetoresistance response at 100 K measured under a bias of *V* = −0.1 V corresponds to a typical MTJ butterfly curve (Figure 4a). The magnetic switching of both LSMO and Co layers is observed, showing coercive fields of around ±85 Oe for LSMO and ±200 Oe for Co. This difference allows for an antiparallel magnetic alignment between the two magnetic electrodes for intermediate magnetic fields. In agreement with previous studies,^22−24^ a lower-resistance state is measured in the antiparallel magnetic configuration when the field is swept, displaying a negative TMR value of 6%, where the TMR is defined as is defined as TMR = , where *R*_AP_ is the resistance in the antiparallel alignment of magnetizations of the electrodes and *R*_P_ is the resistance in the parallel alignment. According to Julliere’s model TMR is governed by the electron spin polarizations of two magnetic electrodes (*P*_1_ and *P*_2_) so that TMR = . This simplified picture does not consider the complexity of the electronic band structure of the ferromagnet, the nature of the tunnel barrier, and the formation of spin-dependent interfacial states^25^ that turn out to be of fundamental importance in the description of tunneling phenomena included in the LSMO/STO/Co MTJ.^13^ Considering that the densities of states for Co and LSMO are both positive,^22^ the inverse TMR at negative bias has been interpreted in terms of interfacial hybridization between Co atoms and the STO barrier, producing a change in the Co tunneling spin polarization. The hybridization at the Co/STO interface due to the formation of covalent bonding of Co and O atoms induces a magnetic moment on the interfacial Ti atoms which are aligned antiparallel to the magnetic moment of the Co layer.^26^ This leads to a negative spin polarization of tunneling across the STO barrier from the Co electrode, inverting the spin polarization at the LSMO/STO interface and hence the TMR signal.

The insertion of a 2 nm thick C_60_ layer acting as a decoupling layer between Co and STO does not change the MR sign, as the hybrid LSMO/STO/C_60_/Co MTJ still has a negative TMR (Figure 4b). While the magnetic switching of LSMO is located at field values similar to those observed for the reference LSMO/STO/Co MTJ, i.e. ±90 Oe, the switching of the Co layer is broader, as a result of the increased Co roughness due to the presence of a C_60_ layer underneath. We emphasize the role of the interfaces between the tunneling layer and the FM metals in favoring a particular spin polarization and electronic character of the tunneling current: the STO/C_60_/Co interface results in a negative polarization in analogy to the STO/Co interface. Since the negative sign of the TMR in LSMO/STO/Co MTJs comes from the inversion of the spin polarization at the STO/Co interface, we conclude that the C_60_/Co interface also features the inversion of the spin polarization and that the electronic structure at the Co surface is modified by the interaction with the C_60_ molecule. This result is in agreement with Moorsoom et al.,^8^ who observed a charge transfer at the Co/C_60_ interface associated with an induced moment in C_60_ molecules antiferromagnetically aligned to the moment of the bulk cobalt and resulting in a behavior analogous to that of Ti atoms at the STO/Co interface. The inversion of polarization at the Co/C_60_ interface was demonstrated also in an AlO_x_ based MTJ^11^ and in a Co/C_60_/Co purely molecular junction,^27^ in agreement with our findings. We also observed that in the case of the C_60_ layer, the TMR value is higher than the TMR reported for MTJs with STO only, reaching nearly 11%.

The TMR effect in both cases decreases with increasing temperature and disappears at nearly room temperature (section SI3), in agreement with most studies performed on other MTJs with LSMO and Co electrodes,^28^ which could be a result of either a decrease in the spin polarization of LSMO at the interface with STO^29^ and/or spin-independent tunneling through impurity levels in the barrier activated upon increasing the temperature.^30^

In addition to the sign, interfaces were found to be critical for the definition of the bias dependence of TMR. In the reference LSMO/STO/Co MTJ, the maximum magnetoresistance value is at a negative bias voltage (*V*_b_ = −0.1 V) and the TMR features a crossover from negative to positive magnetoresistance above the *V*_b_ = +0.2 V threshold value, as we observed in Figure 4c (black balls). This TMR trend is in good agreement with previous works with some minor differences, possibly related to sample quality variations.^31^ This peculiar bias dependence of the reference MTJ has been ascribed to the structure of the DOS of the d band of Co as described by De Teresa et al.^22^ and to the contribution of nonresonant tunneling events trough specific defect states induced by the O vacancies in the barrier.^24^

The insertion of a C_60_ layer results in a different voltage dependence of the TMR, showing only larger negative magnetoresistance over the whole measured bias interval, as shown in Figure 4c (red balls). The bias dependence remains asymmetrical with a maximum absolute value at *V*_b_ = −0.2 V and vanishing TMR for high positive biases. A clue for the interpretation of the bias-dependent behavior of LSMO/STO/C_60_/Co MTJ can be found in our previous *ab initio* calculations on the C_60_ adsorbed on Co.^17^ As was pointed out previously, a magnetic moment, antiferromagnetically aligned to the Co layer, is induced on the C_60_ molecule and correspondingly a decrease of the spin moment of the surface Co atoms beneath the molecule is expected due to hybridization with molecular states. It is worth noting that such calculations refer to C_60_ deposited on a single-crystal Co surface, while in our devices the geometry is reversed due to the deposition of polycrystalline Co on the molecular C_60_ layer. This may induce bias asymmetry effects, similar to those detected in Co/Al_2_O_3_/Co,^31^ where nonsymmetric bias dependence was ascribed to the different crystalline structures of the two electrodes.

**Figure 5:**
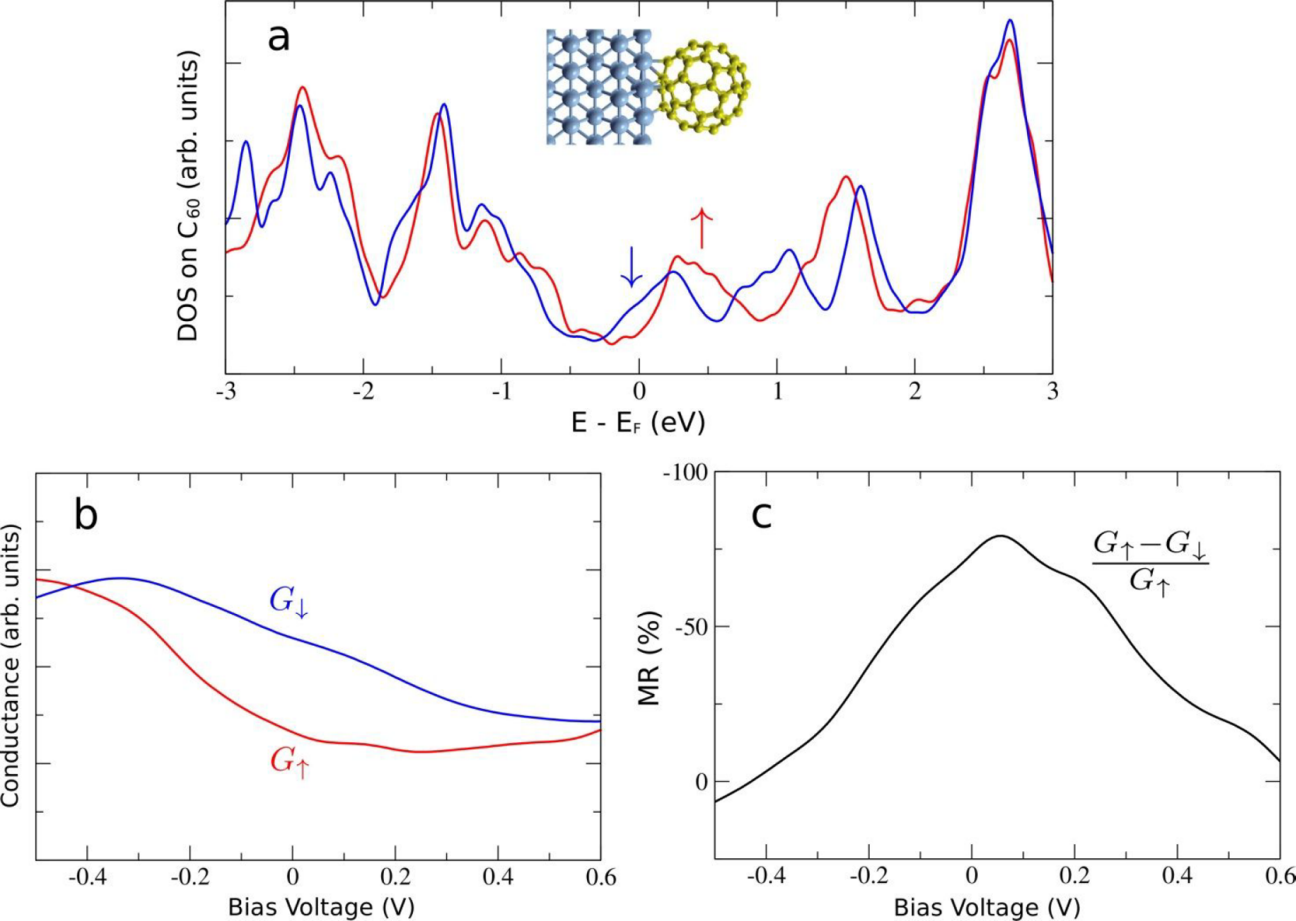
hcp-Co/C_60_ interface with C_60_ in a (5:6)-bond adsorption geometry: (a) spin-resolved PDOS on the C_60_ molecule; (b) spin-resolved conductance calculated from the integrated PDOS; (c) calculated interface TMR (*G*_*↑*_ – *G*_*↓*_)*/G*_*↑*_. Spin-up and -down components in (a) and (b) are plotted in blue and red, respectively. In (b) and (c) a negative/positive voltage corresponds to probing occupied/unoccupied states.

To better clarify the role of Co/C_60_ in the MTJ, we now implement those calculations by evaluating the tunneling probability across the interface. Calculations are carried out using the most stable configuration corresponding to C_60_ adsorbed on epitaxial Co layer in the pentagon–hexagon edge 5:6 bonding as was found in ref (12). Figure 5a presents the spin-resolved DOS projected on a C_60_ molecule (see ref (17) for more details); it is clearly spin polarized at the *E*_F_ value, which would potentially lead to high TMR values due to spin-split hybridized states (coming mostly from the C_60_ lowest unoccupied molecular orbital, LUMO) at the metal–ferromagnetic interface. This finding is in agreement with the experimental observation of a higher TMR signal for a C_60_-based MTJ. In order to better understand the transport properties of the C_60_/Co interface and to make a better connection to the experiment, we compute the spin-resolved conductance (Figure 5b), defined as the corresponding projected DOS (PDOS) integrated over the energy interval [*E*_F_, *eU*] divided by the bias voltage *U*, *G* = *∫*_*E*_F__^*E*_F_+*eU*^PDOS(*E*) d*E*/*U*. This simplified approach allowed is to make tractable our complex problem of spin-polarized transport across the full LSMO/STO/C_60_/Co junction, assuming that all spin dependence comes from the Co/C_60_ interface. Two main assumptions were therefore made: (i) the DOS of LSMO was supposed to be constant in energy (and so could be taken out of the energy integral); (ii) similarly, the tunneling rate of all electronic states across the STO barrier is assumed to be the same and energy-independent. These assumptions, expected to work well at a small bias, can be less justified farther from the Fermi energy (where, for example, an additional minority spin DOS of LSMO appears^32^), which may explain a worse agreement between experimental and theoretical results for increasing bias. Considering the LSMO/STO as a perfect spin-up injector,^13,22^ the TMR of the full junction should depend on the ratio of spin up/down conductances, as represented in Figure 5c. Calculated this way, the TMR curve reproduces experimental data satisfactorily: the calculated TMR is essentially negative and slightly asymmetric with respect to the bias voltage and rapidly decreases with an increasing bias voltage (it is also predicted to become positive at voltages higher than those measured experimentally, *U* > 4 V). Nevertheless, this simulation does not reproduce the position of the TMR maximum, which has been experimentally found at *V* = −0.2 V while calculations place it at positive bias. These discrepancies can be ascribed to the approximations made in our calculations. Indeed, the employed LSMO/STO and Co/C_60_ band structures were based on ideal interfaces without defects and disorder, preventing a precise quantitative comparison with the experimental data collected in polycrystalline samples. Also, considering the absence of TMR inversion and the TMR intensity, the decoupling of STO and Co by the insertion of C_60_ should limit the role of O vacancies in STO in the tunneling process. This could prevent the scattering and the loss of parallel angular momentum conservation^23^ also observed in a defective amorphous STO barrier^33^ and be plausibly responsible for the increase of TMR signal in our devices.

Note that the interfacial hybridization between Co and C_60_ is limited to the first molecular layer, and the derived effects and properties do not depend on the thickness of the organic layer when the devices operate in the tunneling regime.

## Conclusions

In this work, we have shown that the insertion of an ultrathin layer of C_60_ between Co and STO in MTJs strongly affects the TMR response. The substitution in LSMO/STO/Co/tunnel junctions of the Co spin injecting electrode by Co/C_60_ induces a negative sign of the TMR for the whole interval of measured voltage biases, eliminating the well-known effect of the sign change in the prototypical inorganic device, the latter confirmed also in this study on a reference sample. The DFT calculations, performed for an ideal case of C_60_ adsorbed on an epitaxial Co layer, clearly revealed that the negative sign of TMR is induced by the spin-dependent electronic hybridization at the Co/C_60_ interface, which is rather pronounced in the lowest energy adsorption geometry (with C_60_ adsorbed by the pentagon–hexagon 5:6 edge). Notably, the differences between the shapes of calculated and measured voltage dependences of SP are expected to be at least partially caused by the polycrystalline and defective nature of the investigated interfaces, but the presence of more complex and still unknown interfacial effects cannot be ruled out.

Our results demonstrate that hybrid ferromagnetic/molecular interfaces offer versatile routes for tuning of the TMR strength and sign in MTJs, enhancing the choice of spintronic device solutions for logic and memory applications.

## Supporting Information Available

AFM characterization of C_60_ molecules adsorbed on STO, temperature-dependent *I*–*V* characteristics of the LSMO/STO/Co MTJ, and temperature dependence of the magnetoresistance (PDF)
