# Supplementary material for: Spinterface Effects in Hybrid La0.7Sr0.3MnO3/SrTiO3/C60/Co Magnetic Tunnel Junctions
Source: ACS Appl Electron Mater. 2022 Aug 24;4(9):4273–9. doi: 10.1021/acsaelm.2c00300 (PMC9523579; doi:10.1021/acsaelm.2c00300)
Supplement: Supplementary file 1 — el2c00300_si_001.pdf [file el2c00300_si_001.pdf]

Supporting Information

Spinterface effects in hybrid $La_{0.7}Sr_{0.3}MnO_3$ /$SrTiO_3$ /C_{60}/Co magnetic tunnel junctions

Ilaria Bergenti[1], Takeshi Kamiya[2], Dongzhe Li[3] Alberto Riminucci[1], Patrizio Graziosi[1], Donald .A. MacLaren[4], Rajib K. Rakshit [5], Manju Singh [5], Mattia Benini[1], Hirokazu Tada[2], Alexander Smogunov[6], Valentin A. Dediu[1]*

ilaria.bergenti@cnr.it

[1] Institute of Nanostructured Materials ISMN-CNR, Via Gobetti 101, Bologna 40129, Italy

[2] Department of Materials Engineering Science, Osaka University, 1-3, Machikaneyama, Toyonaka, Osaka, Japan, 560-8531

[3] CEMES, Université de Toulouse, CNRS, 29 rue Jeanne Marvig, F-31055 Toulouse, France

[4] SUPA, School of Physics and Astronomy, University of Glasgow, Glasgow G12 8QQ

[5] CSIR - National Physical Laboratory, Dr. K. S. Krishnan Marg, New Delhi, 110012, India

[6]Service de Physique de l'Etat Condensé (SPEC), CEA, CNRS, Université Paris-Saclay, CEA Saclay 91191 Gif-sur-Yvette Cedex, France

S1: C60 molecules adsorbed on STO: AFM characterization

Upon deposition of approximately 2 ML C_{60} (2nm) onto a STO substrate at RT, islands are formed having a typical size of several tens of nanometers in diameter. Figure S1 shows a representative AFM image of the STO substrate (RMS=0.1±0.05 nm)) and of the 2nm C60 on STO. As can be seen from the height profile shown in Fig. S2 , the prevalent height of the islands is 1.5 nm corresponding to nearly double-layer C60 islands. Statistical analysis of surface coverage of the thin films indicated that 95% of the analyzed surface is covered by C_{60}.

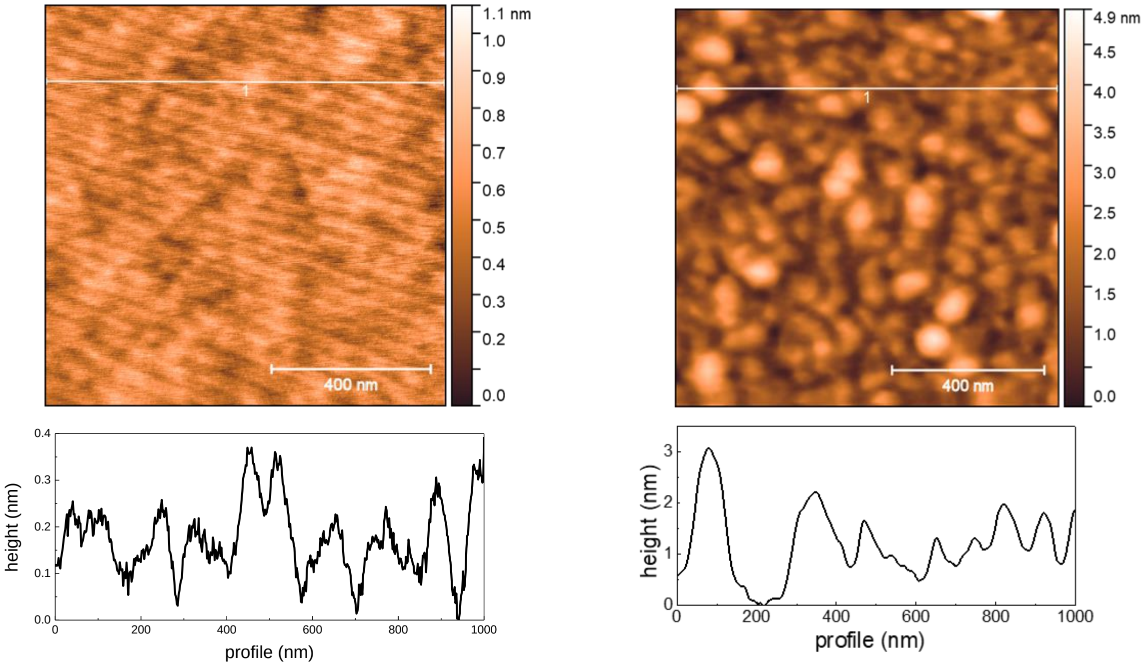

Figure S1: Surface morphology obtained by AFM: STO substrate (right) and 2 nm C_{60} on STO (left). Bottom lines represent line profiles as indicated by the white lines

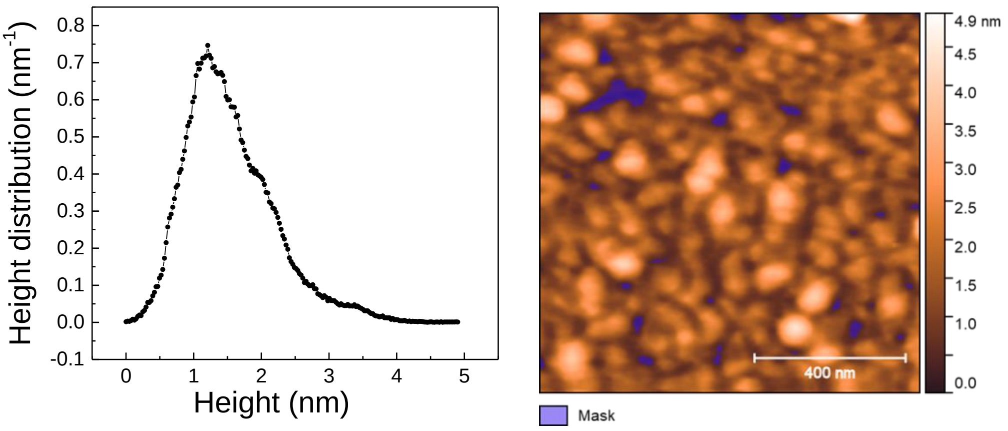

Figure S2: (left) Height analysis for C_{60} islands in the AFM images for 2nm thick c60 layer. (right) Total C60 coverage calculated by masking height below 0.5 nm on the height scales (coverage 98%).

S2: Temperature dependent I-V curves

Temperature-dependent I–V characteristics of LSMO/STO/Co MTJ with a nominal STO thickness of 5nm are shown in fig. S3 All the plots depict nonlinear and quasi-symmetric I–V characteristics. The R(T) curve exhibits a smooth increase of the resistance on lowering temperature compatible with direct tunneling transport. However, R(T) exhibits a peak with a maximum at about 200 K(fig S3, blackballs) that seems to be a common feature in LSMO/STO/Co MTJs and whose origin is not well established yet.[1]. In case of C_{60} insertion, the R(T) exhibits a purely insulating behavior, without traces of the LSMO contribution. The change in R(T) in case of C60 insertion is also an indication of the effectiveness of the molecular layer in transport, excluding the metal penetration through the molecular layer and the subsequent molecular damage that typically result in behavior characteristic of electronic shorts

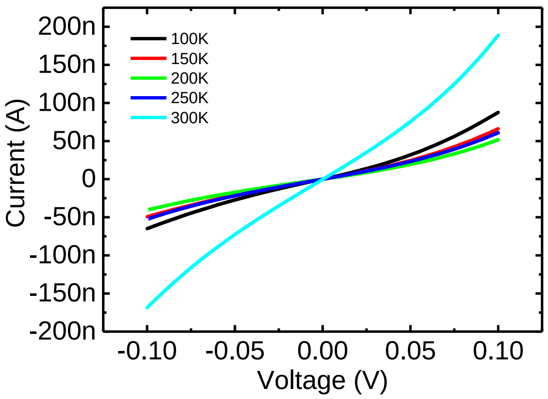

Figure S3: I-V measurements for the reference sample LSMO(15nm)/STO(5nm)/Co(50nm), without applying an external magnetic field.

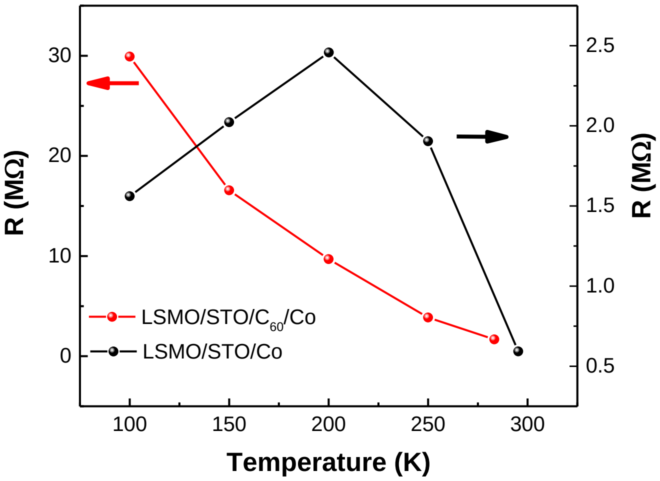

Figure S4: Temperature dependence of the MTJ resistance

S3: Temperature dependent MR

The temperature dependence of the TMR in the LSMO based junctions decays rapidly and vanishes below the Curie temperature of LSMO layer that is close to 320K for such 15 nm thick layer[2]. Since tunneling reflects mainly the properties of the electrode/barrier interface, this behavior has been ascribed to a premature loss of the spin polarization at interfaces. This is consistent with the weakening of the ferromagnetism of LSMO[3].

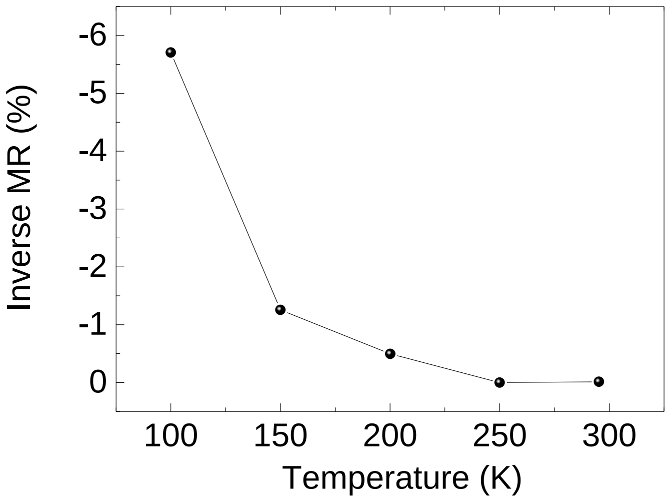

Figure S5: Temperature dependence of the MR resistance for LSMO/STO/Co MTJ measured at bias V=-0.1 V

(1) Sun, J. Z.; Roche, K. P.; Parkin, S. S. P. Interface Stability in Hybrid Metal-Oxide Magnetic Trilayer Junctions. *Phys. Rev. B* **2000**, *61* (17), 11244–11247. https://doi.org/10.1103/PhysRevB.61.11244.

(2) Garcia, V.; Bibes, M.; Barthélémy, A.; Bowen, M.; Jacquet, E.; Contour, J.-P.; Fert, A. Temperature Dependence of the Interfacial Spin Polarization of La2/3Sr1/3MnO. *Phys. Rev. B* **2004**, *69* (5), 052403. https://doi.org/10.1103/PhysRevB.69.052403.

(3) Park, J.-H.; Vescovo, E.; Kim, H.-J.; Kwon, C.; Ramesh, R.; Venkatesan, T. Magnetic Properties at Surface Boundary of a Half-Metallic Ferromagnet La0.7Sr0.3MnO3. *Phys. Rev. Lett.* **1998**, *81* (9), 1953–1956. https://doi.org/10.1103/PhysRevLett.81.1953.
